# Spatio-temporal divergence and influencing factors of agritourism integration development in Xinjiang, China

**DOI:** 10.1038/s41598-023-46806-5

**Published:** 2023-11-08

**Authors:** Yaping Peng, Weizhong Liu, Changjiang Xiong

**Affiliations:** 1 College of Economics and Management, Xinjiang Agricultural University, Urumqi, 830052 Xinjiang China; 2 Institute of Finance and Economics, Shanghai University of Finance and Economics, Shanghai, 200433 China

**Keywords:** Agroecology, Sustainability

## Abstract

Exploring the coupling and coordination relationship between agriculture and tourism industry plays an important role in formulating differentiated integration development policies and promoting regional economic development. We adopt 14 prefectures and cities in Xinjiang, China as examples, and use a coupling coordination model and generalized method of moments estimation to explore the spatial differentiation characteristics and influencing factors between agriculture and tourism industries in various prefectures and cities in Xinjiang from 2009 to 2018. The results indicate: (1) The combined development levels of agriculture and tourism in Xinjiang exhibited a similar development trend over the period, and despite fluctuations in individual years, the overall trend is still increasing. (2) The coupling degree and coordination of agriculture and tourism in various regions presented significant spatial differentiation characteristics. (3) Human capital, residents’ consumption level, and coupling coordination degree are positively correlated, while service capacity, government support, industrial foundation, and market supply and coupling coordination degree are significantly negatively correlated. Finally, based on our results, we present corresponding suggestions on how to improve the level of integrated development of agriculture and tourism in Xinjiang.

## Introduction

The countryside has multiple functions such as life, culture, and ecology, and together with the towns and cities, constitutes the space for human activities on the basis of mutual promotion and coexistence. Since reform and opening up, China’s agriculture and rural areas have made remarkable achievements, and farmers’ incomes and living standards have been rising. However, they also face problems such as weak agricultural competitiveness, imbalance in the to-go allocation, and insufficient efficiency of agricultural inputs and outputs. In 2017, China proposed the implementation of the rural revitalization strategy to improve quality and efficiency of agriculture. However, for most Chinese villages, agri-tourism integration is a powerful tool for rural revitalization and a new type of industrial innovation. agri-tourism integration leads to transfer of land, capital, and other factor endowments, promotes the development of agricultural socialized services, and ultimately realizes the upgrading and adjustment of industrial structure. As a multi-ethnic agglomeration area in China, Xinjiang is particularly rich in natural, cultural, and ecological resources, the integrated development of agriculture and tourism industry is a realistic option to transform the resource advantages of multi-ethnic agglomeration areas into industrial advantages. Excavating the resource endowment and industrial characteristics of leisure agriculture and rural tourism in Xinjiang, promoting the extension of the agricultural industry chain, enhancing the industry chain, broadening the income chain, and realizing the healthy development of agriculture are of practical and theoretical significance to promote the strategy of revitalization of Xinjiang’s countryside and realize the goal of common prosperity.

We adopt 14 prefectures and cities in Xinjiang as an example to explore the spatial differences and influencing factors between agriculture and tourism industries in various prefectures and cities from 2009 to 2018. We provide theoretical support for the promotion of the healthy development of agriculture-tourism integration. The comprehensive evaluation values of agriculture and tourism in all cities and towns in Xinjiang are on an upward trend, and the overall trend is positive. In general, the level of agricultural and tourism development is better in southern and Northern Xinjiang, respectively. The levels of development vary from region to region, but the phenomenon of “polarization” persists. Regarding industrial integration, the degree of coupling and coordination between the agricultural and tourism industries in all regions exhibit significant spatial differentiation, and the degree of integration of various factors and resources in the southern region needs to be improved. Among the factors influencing the coordinated development of the integration of agriculture and tourism, human capital and residents’ consumption level have a positive influence, while service capacity, government support, industrial base, and market supply have a negative influence.

The contributions of this study are mainly as follows. First, we propose a logical framework for the formation of the integration of agriculture and tourism, which compensates for gap in the previous theoretical research. Second, the factors affecting the coupling and coordination degree of agriculture and tourism are refined into service capacity, human capital, government support, residents’ consumption level, industrial foundation, and market supply. A new methodology was also adopted to empirically test the factors affecting the coupling and coordination degree of agriculture and tourism, which provides a new perspective for further understanding how to push the coordinated development of agriculture-tourism integration in Xinjiang.

## Literature review

The current research on agritourism integration mainly focuses on the concept and connotation, mode, measurement of integration, and so on. To clarify the research results of agritourism integration, we investigate the relevant literature from four aspects: connotation, mode, measurement, and the effect of agritourism integration (see Table 1).

**Table 1:** Overview of major domestic and international studies on integrated agri-tourism development.

Classification	Author	Method	Subject	Main points
Concepts	Marques (2006)	Interview	Wine region of northern Portugal	Agritourism is a category of rural tourism, but with a different focus. Rural tourism focuses on the natural environment, while agritourism focuses on farmer participation
	Zhang(2009)	Theoretical analysis	China	Agri-tourism integration is a new type of agricultural service industry formed by the intersection of the primary and tertiary industries
	Rainey et al. (2010)	Factor analysis	Arkansas Farm Operators and Landowners	Agritourism refers to all activities and industries in which the basic elements of the two industries are integrated to enhance the profitability of agribusinesses and farmers
	Blekesaune et al. (2010)	Case study	Norway	Agritourism and rural tourism are similar in that agritourism is only a part of rural tourism
	Ye (2014)	Theoretical analysis	China	Agri-tourism complex is actually a product of a high degree of integration between agriculture and tourism
	Vasta et al. (2015)	Interview, questionnaire	Eastern Pacific Coast of the United States	Agri-tourism integration is a combination of commercial tourism and agriculture, and a new form of agricultural production and operation
	Francesca et al. (2016)	Case study	Campania region, Italy	Role of green buildings for agritourism should be highlighted
Modes	Liu (1998)	Case study	China	Urban agriculture type, agricultural fairs, and other models
	Busby and Rendle(2000)	Case study	New Zealand, Europe, North America	“Company + farmers,” “farmers + farmers,” “share development system” and other models
	Phillipet et al*.*(2010)	Theoretical analysis	Scotland	Demonstration of agritourism, authentic agritourism, non-working farm agritourism and other models
	Liu et al. (2013)	Theoretical analysis	China	Youth agricultural science and technology science park, leisure landscape farm, agricultural culture tourism creative industrial park, and other models
	Easton (2017)	Case study	Tuscany	Science education tourism landscape and ultra-modern consumer landscape models
	Murthy (2010)	Multiple linear regression	India	Integration of agri-tourism can contribute to the growth of regional economic benefits
	Zhang et al. (2015)	Coupled coordination evaluation model	Zhangjiajie, Hunan, China	Agriculture is lagging behind tourism development, and systemic incoherence hinders sustained regional economic development and tourism competitiveness
	Xia et al. (2016)	Vector autoregressive model	China	Integration of tourism and agriculture is a relationship that promotes each other
	Wang (2018)	Principal component analysis, coupled coordination model	Shanxi Province, China	Coupling and coordination of the two systems of agriculture and tourism in Shanxi Province gradually shifted from an initial serious disorder to an initial coordinated development
	Qiu et al. (2021)	Coupled coordination evaluation model	Henan Province, China	Henan’s agricultural and tourism industries interact with each other, with increasing levels of integrated development and coordination
	Gu et al. (2021)	Data envelopment analysis	Yellow River Basin, China	Overall high level of efficiency of agri-tourism integration in the Yellow River Basin region
Impacts	Davies et al. (1992)	Case study	Wales Farm, UK	Agritourism can contribute to the transformation of the agricultural industry while also increasing national tourism revenues
	Fang (2013)	Entropy method	South Dongting Lake Area, China	Integration of agriculture and tourism can create significant social and economic effects
	Anderson (2018)	Value chain analysis	Tanzania	Agri-tourism integration can boost regional economic development and alleviate regional poverty
	Lane et al. (2018)	Interview, gap analysis	Agritourism operators on small farms	Agri-tourism not only meets the diversified needs of tourists, but also absorbs surplus rural labor, thus generating an industrial economic multiplier effect
	Zhang (2019)	Theoretical analysis	China	Agritourism integration is a breakthrough in rural revitalization and can promote the modernization of agriculture
	Charles et al. (2020)	Semi-structured interviews, questionnaire method	Saint Lucia	Agri-tourism can extend the agricultural industry chain and accelerate the transformation, upgrading and industrialization of agriculture

### Connotation of agritourism integration

The development of agritourism integration started earlier in foreign countries. Results on the connotation of agri-tourism integration are mostly from the perspective of industrial boundaries^1,2^, integration of new business forms^3,4^, resource penetration^5^, sustainable development theory^6^, and other perspectives. The relevant findings are based on empirical research conducted in different countries on the one hand, and the dynamic evolution of the connotation of agri-tourism integration in different historical periods on the other. Chinese scholars have broadened the connotation of agritourism integration to a new theoretical scope and presented new concepts on the basis of foreign studies. For example, Zhang and Chen regarded agri-tourism integration as a new type of agricultural service industry formed by the intersection of primary and tertiary industry^7^. Ye and Zhou offered the new concept of agri-tourism complex, which is actually the product of a high degree of integration between agriculture and tourism^8^.

### Model of agritourism integration

The model of agritourism integration development is also becoming increasingly widespread. Early foreign scholars mostly studied the model from the perspective of farmers, such as the traditional “company + farmers,” “farmers + farmers,” and other modes^9^. However, with the deepening of research and the change of demand, scholars are beginning to focus on experiential tourism models from the perspective of tourists, such as “demonstration agritourism,” “non-working farm agritourism,” and so on^10^. Nowadays, from China’s empirical research, the development of postmodern tourism has made tourism and leisure an important means of acquiring knowledge, expanding horizons, and developing skills, and led to a number of novel modes of nurturing value: science and education tourism landscape mode^11^, the youth agricultural science and technology science park mode^12^, and the agricultural exposition-type mode^13^, among others.

### Measurement of agritourism integration

For the healthy development of agritourism integration, relying only on exploring the connotation and mode of its development is not sufficient; it is also necessary to adopt scientific quantitative analysis methods to explore its development level. The closely related literature on the level of agritourism integration development is categorized into three main types: (1) Literature centered around multivariate linear regressions to assess the relationship between agritourism integration and the variables of interest^14^. (2) Literature focused on measuring the level of agritourism integration development around coupled and coordinated evaluation models^15–17^. (3) Literature that utilizes data envelopment analysis (DEA) to measure the level of regional agritourism integration efficiency^18^.

### Effect of agritourism integration

In terms of the effect of agritourism integration at home and abroad, some scholars have verified that it has a promoting effect on economic growth^19,20^. Agritourism integration can contribute to economic growth and farmers’ incomes^21–23^. Conversely, agri-tourism integration can promote industrial transformation and upgrading, and the process is actually that of interactive development of agriculture and tourism to produce new industries^24–27^. However, some scholars argue that the development process of agri-tourism integration has brought economic effects and caused impacts on the ecological environment in a specific region^28^. Undeniably, scholars at home and abroad have reached a consensus on the effects produced by agri-tourism integration.

In summary, existing studies have explored the connotation, mode, measurement, and impact effect of agri-tourism integration to a certain extent, which has important reference value for this study. Additionally, combined with the comparative analysis of existing studies, we argue that the following aspects warrant attention in the process of further promoting the research on the topic of agri-tourism integration. Regarding research perspective, the geographical space chosen in previous studies is relatively diverse, mostly from the national level, provincial level, or developed regions, but fewer studies focus on ethnic areas of Northwest China. Regarding research methodology, there is a lack of dynamic analysis using panel data and measurement indicators applicable to ethnic regions. In view of this, we take Xinjiang, an ethnic region in China, as a typical case study, to analyze the role mechanism and level of coupled and coordinated development of agriculture and tourism industries. We reveal the spatial differentiation characteristics and influencing factors of agriculture-tourism integration, and present the path to promote such integration in Xinjiang.

## Formation logic of the integrated development of agriculture and tourism

**Figure 1:**
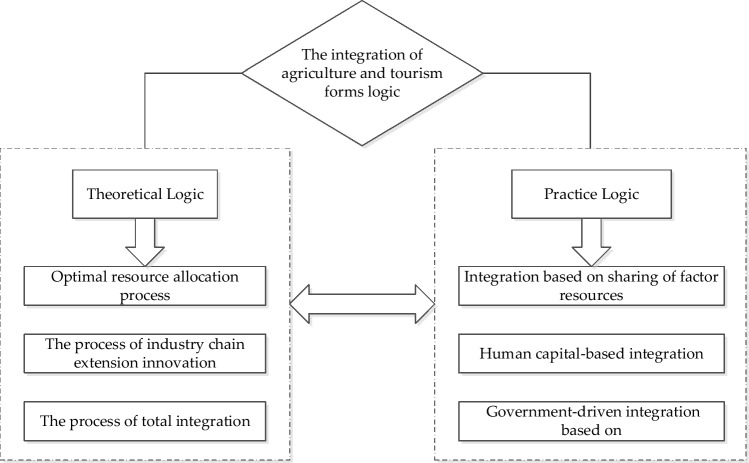
Schematic diagram of the formation logic of agri-tourism integration.

Agritourism integration is the cross-pollination of agriculture and tourism, leading to the gradual formation of new models^29^. Its essence is the mutual extension and integration between the two; agriculture provides high-quality tourism resources for the tourism industry, tourism provides a new direction for the development of agriculture. The mutual penetration of the two can achieve the optimal allocation of resources, and ultimately the sharing of factor resources. This study elaborates the formation mechanism of agriculture-tourism integration from two aspects of theoretical and practical logic; the specific framework is illustrated in Fig. 1.

### Theoretical logic

The integration of the two industries needs to rely on agricultural production and ecological landscapes and promote the economical use of resources and energy through rational planning and utilization of unused land resources. Gradual spillover of developed knowledge and management systems from the tourism sector to the agricultural sector, which can continue to improve agro-ecological efficiency through a process of resource optimization and technological integration^30^. The integration of agriculture and tourism is the process of industrial chain extension and innovation. Industrial integration requires a certain degree of correlation between industries, and it is necessary to accelerate the optimization and upgrading of the structure of the agricultural industry, promote the extension of the agricultural industry chain, and innovate the mode of agricultural production and management. For the tourism industry, under the influence of economic, technological, cultural, and conceptual factors, new tourism products are bound to emerge, and the structure of the industry chain model will also change^31^. Through the integration and development of the two, the industrial system of agriculture and the value-added of agricultural products can be improved. Tourism can also produce new products in line with the market orientation, so that the tourism industry chain can be extended and innovated. The integration of agriculture and tourism is a comprehensive integration process of mutual crossing and penetration, so it must be integrated from multiple dimensions, namely resource, technology, function, and market integration.

### Practice logic

In practice, the core goal of agri-tourism integration is the integration and sharing of various factor resources between the two, including integration based on factor resource sharing, on human capital, and on government promotion. First, integration based on element resource sharing. Xinjiang is located in the Eurasian hinterland, with unique agricultural cultivation resources and excellent tourism resources. In the context of industrial integration, resource elements of Xinjiang’s two major industries should be fully integrated, that is, multi-participation, multi-mode and multi-line promotion, so that all tourism stakeholders can and should obtain benefits through tourism, so that everyone can share the economic dividends brought by tourism. Second, integration based on human capital. The level of tourism human capital can affect the quality of tourism services, the dissemination of tourism image, and so on. The integration of tourism human capital is the foundation for the integration of agriculture and tourism, which can improve the efficiency of tourism economy^32^. Third, government-driven consolidation. The rapid development of tourism is closely linked to national strategies and policies, mainly in terms of active mobilization of relevant resources and activation of potential tourism markets. China’s tourism industry has always been centered on the “government-driven market-first development model”^33^, which is mainly manifested at two levels: (1) through policy documents and industrial planning to guide the benign development of the agricultural and tourism industries. (2) To improve the rural infrastructure and continuously upgrade the level of public services, leading to the expansion of the rural tourism market.

## Study design

### Study object

Xinjiang is located in the hinterland of the Eurasian continent and is the core area of the Silk Road Economic Belt. As of the end of 2018, agriculture, forestry, and fisheries account for 29.82% of Xinjiang’s GDP while tourism industry accounts for 21.15%. Agriculture is still the main industry on which Xinjiang farmers rely for survival, and tourism is a strategic pillar industry for economic development. However, there is still an obvious gap in the development of tourism among various prefectures. Data from 2018 reveal six prefectures in Xinjiang with domestic tourism income exceeding 10 billion yuan: Urumchi (65.25 billion, which ranked the highest), Ili (64.31 billion), Changji (50.795 billion), Altay (22.233 billion), and Bayingol (11.753 billion). Kizilsu had the lowest, with 872 million yuan. For inbound tourism income, Ili ranked top with 1.9977 million people, followed by Urumqi (331,200), Altay (235,200), Turpan (127,700), and other prefectures with fewer than 100,000. The lack of integration of agriculture and tourism is also a key constraint to development in arid areas, as well as the widening of the gap in tourism development, in addition to the impact of geographic location, transportation conditions, the basis for development, and other factors.

### Establishment of index system

The index of agricultural diversification, cultivated area, and total power of agricultural machinery^17,22^ has been adopted to evaluate the level of regional agricultural development. The regional development level of tourism is evaluated from the aspects including number of inbound tourists, scenic spots at 4A-level and above, travel agencies, and so on^22,34^. According to existing research, the variability of regional development makes some of the measurement indicators incompatible, and the indicators used for micro-level studies in particular are subject to uncertainty. Considering the lack of relevant statistics for the indicators of individual tourism industries in the 14 prefectures of Xinjiang, we established an evaluation index system for the integration of agriculture and tourism (Table 2) according to the overall logic of the industry and the availability and comparability of data, aiming to measure the level and effectiveness of the integration of agri-tourism.

**Table 2:** Evaluation index system for agriculture-tourism integrated development.

Project	Index	Unit	Weight
Agricultural development level (A)	Gross output value of agriculture (A_1_)	10,000 Yuan	0.218
	Agricultural acreage (A_2_)	1000 hectares	0.289
	Per capita net income of rural population (A_3_)	Yuan	0.075
	Agricultural personnel in rural area (A_4_)	Person	0.215
	Total agricultural machinery power (A_5_)	Kilowatt	0.204
Development level of tourism (B)	Domestic tourists (B_1_)	10,000 people	0.107
	Inbound tourists (B_2_)	10,000 people	0.246
	Domestic tourism income (B_3_)	10,000 Yuan	0.166
	International tourism income (B_4_)	10,000 Dollars	0.255
	The number of tourism agencies (B_5_)	One	0.162
	The number of star-rated hotels (B_6_)	One	0.063

### Study method

1. Determination of the weight of indexes.
  The entropy method determines the weight of indexes by analyzing the correlation degree and information among indexes, avoiding the deviation caused by subjective influence to some extent.
  First, the data is normalized to obtain the normalized matrix *B*_*ij*_, and then calculate the entropy *S*_*i*_ with the following method:
  1 $$S_{{_{i} }} = \frac{{ - \left( {\sum {C_{i} \times InC_{i} } } \right)}}{Inn},\;C_{i} = {{B_{i} } \mathord{\left/ {\vphantom {{B_{i} } {\sum {B_{i} } }}} \right. \kern-0pt} {\sum {B_{i} } }}$$
  where *C*_*i*_ is the proportion of the *i*th index in the evaluation samples, *n* is the number of evaluation samples, and *S*_*i*_ is the entropy value. Finally, the index weight *W*_*i*_ is calculated based on the following formula:
  2 $$W_{i} = ({{1 - S_{i} )} \mathord{\left/ {\vphantom {{1 - S_{i} )} {\sum ( }}} \right. \kern-0pt} {\sum ( }}1 - S_{i} )$$
2. Establishment of the synthesizing evaluation function of agriculture
  3 $$F(x) = \sum\limits_{i = 1}^{n} {w_{i} } M_{ij}$$
  where *i* is the number of agricultural projects (*i* = 1, 2, …, *n*), *W*_*i*_ is the index weight, and *M*_*ij*_ is the standardized value of the *i*th indicator of agriculture in year *j*. The larger the result, the better the condition of agriculture.
3. Establishment of the synthesizing evaluation function of tourism
  4 $$G(y) = \sum\limits_{i = 1}^{n} {w_{i} } N_{ij}$$
  where *i* is the number of agricultural projects (*i* = 1, 2, …, *n*), *W*_*i*_ is the index weight, *N*_*ij*_ is the normalized value of the *i*th indicator of tourism in year *j*. The larger the result obtained, the better the condition of the tourism, otherwise it is not.
4. Coupling model. The degree of coupling between systems is reflected with the help of the coupling degree function, which is calculated as follows:
  5 $$C = \sqrt {f(x) \cdot f(y)} /(f(x) + f(y))$$
  where *C* is the coupling degree, lying between [0, 1]. According to existing studies^18^, the coupling degree value is divided into four intervals, (Coupling degree standard: low level (0 < C ≤ 0.3), antagonistic (0.3 < C ≤ 0.5), breaking-in (0.5 < C ≤ 0.8), high-level stage (0.8 < C ≤ 1).) with each representing the corresponding coupling state.
5. Finally, the coupled coordination degree model is constructed, which is defined as in (6):
  6 $$\left\{ {\begin{array}{*{20}l} {D = \sqrt {C \times T} } \hfill \\ {T = \alpha f(x) + \beta g(y)} \hfill \\ \end{array} } \right.$$
  The two systems are intersecting, infiltrating, and acting equally with each other; hence, $\alpha$ = $\beta$ = 0.5. According to the related studies^18,34^, the coordination degree is divided into three major categories and 10 levels. (Coupling coordination standard: Recession caused by disorder: Extreme [0,0.09], severe [0.10, 0.19], moderate [0.20, 0.29], mild [0.30, 0.39]. Over-development: on the verge of disorder [0.40, 0.49], barely coordination [0.50, 0.59]. Coordinated development: primary [0.60, 0.69], intermediate [0.70, 0.79], good [0.80, 0.89], excellent [0.90, 1]).

### Data sources

Based on panel data from 14 prefectures and cities in Xinjiang, we adopt 2009–2018 as the time frame to measure the integration level of agriculture and tourism . Data are mainly derived from *Xinjiang Statistical Yearbook*, Statistical Bulletins of Municipalities, and reports on the relevant government websites of Municipalities.

## Results and analysis

### Comprehensive development level of agriculture and tourism

**Figure 2:**
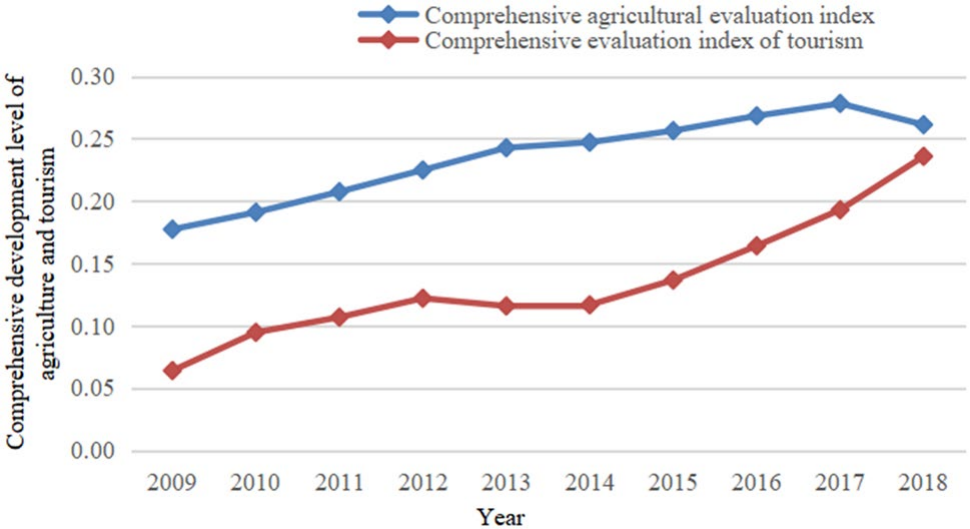
Xinjiang agriculture and tourism composite evaluation index.

The combined development levels of agriculture and tourism in Xinjiang from 2009 to 2018 have generally exhibited an upward trend (See Fig. 2). The comprehensive development level of agriculture is higher than that of tourism. This is because Xinjiang is an important plantation base and livestock base in China, and the development of agricultural technology has further enhanced its comprehensive agricultural development. Although the tourism industry has generally displayed an upward trend, the overall trend has been slow, and has been greatly affected in individual years. This is because Xinjiang is limited by factors such as geographic location, infrastructure, and demographic characteristics, which restricts the enhancement of the comprehensive development level of tourism industry to a certain extent.

Regarding time, (1) from 2009 to 2012, the comprehensive development levels of both agriculture and tourism have risen slowly and maintained a positive momentum. (2) From 2013 to 2014, the comprehensive development levels of agriculture slowed while those of tourism displayed a downward trend. This is mainly owing to the obvious short-board effect of the tourism industry, the peak season is too short and the off-season is long, and the comprehensive benefits of tourism are easily affected, leading to a clear downward trend. (3) from 2015 to 2018, the comprehensive tourism development level exhibited a rapid recovery in growth. To further cultivate tourism as a strategic pillar industry, the Xinjiang government has introduced various measures to improve the tourism industry and product systems. A regional tourism segment has been built, which has led to a climax in the development of Xinjiang’s tourism industry after a brief downturn. However, it is evident from the combined level of agricultural and tourism development in 2018 that the growth of tourism has not fully driven the development of agriculture, that is, the role of tourism in driving agricultural development has not been fully realized.

## Spatial differentiation characteristics between coupling and coupling coordination degree

### Spatial differentiation of coupling degree

**Figure 3:**
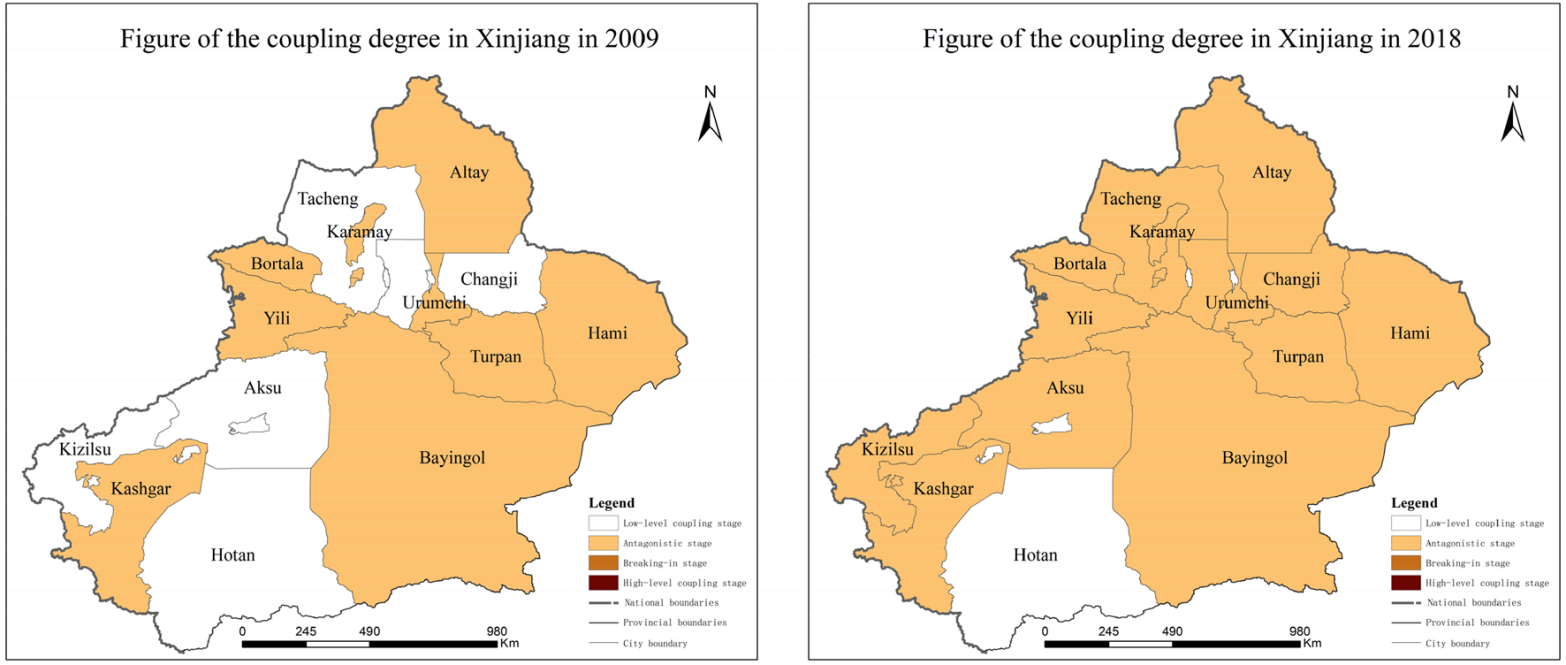
Variation of coupling degree between agriculture and tourism in Xinjiang (2009–2018).

The 14 prefectures and cities in Xinjiang showed spatial characteristics of asynchronicity, regional differences, and regional polarization regarding the coupling degree of agri-tourism integration from 2009 to 2018 (Fig. 3). Overall, the degree of coupling displays a trend of continuous optimization, and the northern regions are better than the southern regions. Specifically, the Northern Xinjiang region is in the “antagonistic” stage, in which the space in this stage of the Northern Xinjiang region as a whole is gradually expanding. In particular, Tacheng and Changji have made full use of their unique folk culture, special agricultural resources, and unique tourism resources to significantly improve the level of integration of the agricultural and tourism industries. In the Southern Xinjiang region, with the exception of Hotan, the rest of the region has reached the “antagonistic” stage. Aksu and Kezhou have vigorously promoted the optimization and adjustment of their agricultural and tourism structures, making use of their rich and distinctive rural resources and developing their planting and tourism industries according to local conditions. The Hotan region as a whole is still at the “low level of coupling” stage. The possible reasons for this are its weak infrastructure and lack of human resources, which constrain the development of agri-tourism integration.

### Spatial differentiation characteristics of coupling coordination degree

**Figure 4:**
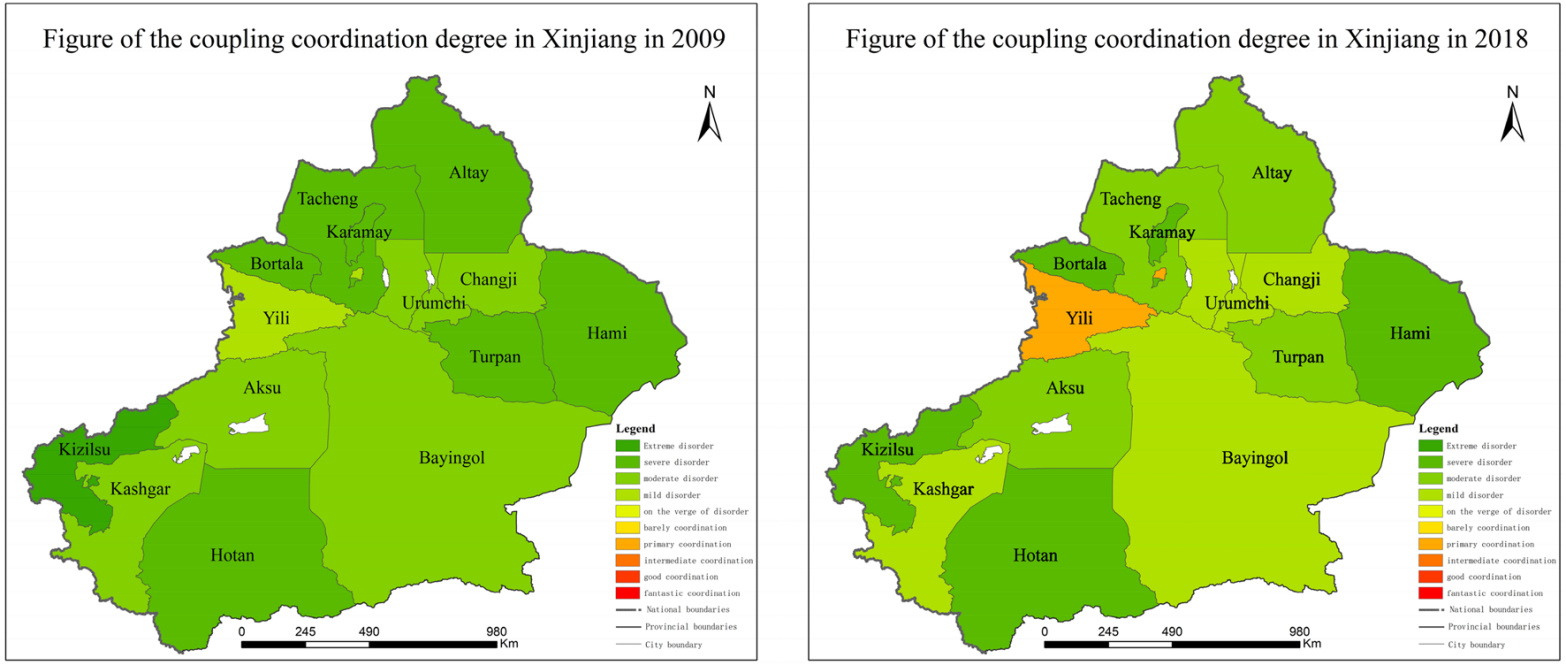
Variation of coupling coordination degree between agriculture and tourism in Xinjiang (2009–2018).

From 2009 to 2018, the coupling coordination degree of agricultural-tourism integration was relatively low, but the overall trend was mainly a steady increase (Fig. 4). Overall, the degree of coupling coordination changes from “failure to decline” to “coordinated development,” and is higher in the northern regions than the southern regions. The Northern Xinjiang region is mostly at the “mildly dysfunctional” or “moderately dysfunctional” stage. Ili Prefecture has the most obvious upward trend, crossing a total of three levels and moving into the initial coordination type, ranking first in the whole territory. The main reason is that Ili Prefecture has a good agricultural foundation and a unique location advantage, and the integration of agriculture and tourism development has been effective. By contrast, the level of coupling coordination between the cities of Karamay and Hami is low and in a state of “serious dissonance.” The possible reasons for this are that regarding agriculture, Karamay is constrained by geomorphological, climatic and soil conditions, and regarding tourism, Karamay is a typical resource-based city, with insufficient natural and human resources, making it difficult to develop a combination of advantages. Hami is similarly limited in agriculture and tourism resources, and the state of agri-tourism integration is poorer and lower. The level of coupling coordination in Southern Xinjiang is mostly at the “moderate dissonance” or “serious dissonance” stage. On the whole, the degree of coordination of agriculture and tourism integration in Kashgar is relatively high, which is “mildly disordered.” The possible reason is that Kashgar region has continued to optimize the industrial layout and strengthen its own agricultural-tourism resource advantages in recent years, making the whole agricultural industry chain empowered rural tourism. The Kezhou and Hotan regions are in a stage of “serious dislocation.” Specifically, these have typical continental arid climates, with extreme drought and low rainfall, low vegetation cover, fragile ecological environments, and relatively lagging agricultural development, so that their coupling degree of harmonization is in a relatively poor state.

### Comparative analysis of mean value of coupling coordination degree

For the average evaluation values and coupling coordination degree of the two systems, the lagging tourism industry is the main factor restricting the level of coupling and coordination development in various states (See Table 3). Except Urumqi, all other prefectures exhibit such lag, indicating that the contribution of tourism to agriculture is obviously less than that to tourism. The reason is that although the abundant tourism, historical and cultural, and ecological and environmental resources in Xinjiang contribute to the development of tourism so as to help poor people overcome poverty, the important economic status and role of tourism industry have been neglected, and the exploitation of natural resources is at a low level. Urumqi displays a lag in agricultural development. As the capital of Xinjiang Uygur Autonomous Region, as well as the radiation and distribution center of tourism industry in Xinjiang, the development of tourism in Urumqi is rapid with abundant tourism resources around the city. In contrast to the weaker development of the agricultural industry, the arable land area of Urumqi is only 73,766.91 hectares as of 2018, which fundamentally and seriously limits the upward mobility of agriculture, resulting in the phenomenon of lagging agricultural development type. From the above results, the level of development of agri-tourism integration has not been synchronized across the states.

**Table 3:** Mean values of coupling coordination degree between agriculture and tourism in each prefecture of Xinjiang (2009–2018).

Prefecture	Evaluation value of agriculture	Evaluation value of tourism	Coupling degree	Coupling coordinated degree	Coordination type	Main restriction factor
Urumchi	0.077	0.388	0.374	0.291	Moderate disorder	Lagging of agriculture development
Karamay	0.047	0.034	0.492	0.139	Severe disorder	Lagging of tourism development
Turpan	0.092	0.061	0.482	0.192	Severe disorder	Lagging of tourism development
Hami	0.071	0.027	0.448	0.148	Severe disorder	Lagging of tourism development
Changji	0.349	0.083	0.358	0.278	Moderate disorder	Lagging of tourism development
Ili	0.733	0.430	0.465	0.518	On the verge of disorder	Lagging of tourism development
Tacheng	0.315	0.025	0.253	0.207	Moderate disorder	Lagging of tourism development
Altay	0.110	0.094	0.497	0.227	Moderate disorder	Lagging of tourism development
Bortala	0.099	0.025	0.391	0.155	Severe disorder	Lagging of tourism development
Bayingol	0.254	0.066	0.401	0.253	Moderate disorder	Lagging of tourism development
Aksu	0.386	0.034	0.271	0.238	Moderate disorder	Lagging of tourism development
Kizilsu	0.052	0.009	0.335	0.101	Severe disorder	Lagging of tourism development
Kashgar	0.522	0.060	0.299	0.296	Moderate disorder	Lagging of tourism development
Hotan	0.191	0.015	0.262	0.163	Severe disorder	Lagging of tourism development

## Factors influencing the coupled and coordinated development of agriculture and tourism in Xinjiang

### Model construction

To further explore which factors affect the degree of coupled coordination of agritourism integration in Xinjiang, we draw on existing research results and construct an econometric model. Considering the dynamic nature of coupling coordination, that is, the prior period coupling coordination affects that of the current period, a dynamic panel model is selected for empirical analysis:

7 $${\text{Y}}_{{{\text{it}}}} = {\upalpha }_{0} + {\upalpha }_{1} {\text{Y}}_{{{\text{it}} - 1}} + {\upalpha }_{2} {\text{se}}_{{{\text{it}}}} + {\upalpha }_{3} {\text{ca}}_{{{\text{it}}}} + {\upalpha }_{4} {\text{gov}}_{{{\text{it}}}} + {\upalpha }_{5} {\text{con}}_{{{\text{it}}}} + {\upalpha }_{6} {\text{in}}_{{{\text{it}}}} + {\upalpha }_{7} {\text{ma}}_{{{\text{it}}}} + {\upbeta }_{{\text{i}}} + {\upvarepsilon }_{{{\text{it}}}}$$

where *i* denotes each region (*i* = 1, 2, 3,…, 14) and* t* represents time (*t* = 2009, 2010, 2011,…, 2018). *α* is the coefficient matrix, ${\beta }_{i}$ denotes the fixed effect coefficient. The ${Y}_{it}$ explanatory variable represents the degree of coupled coordination between agriculture and tourism in Xinjiang, and and the explanatory variables ${se}_{it}$,${ca}_{it}$, ${gov}_{it}$, ${con}_{it}$, ${in}_{it}$, and ${ma}_{it}$ represent service capacity, human capital, government support, residents’ consumption level, industrial base, and market supply, respectively.

The inclusion of one-period lagged terms of the explanatory variables in the above model produces biased and inconsistent fixed or random effects estimates for ordinary panels when conducting regressions. Therefore, we choose the generalized method of moments (GMM) estimation model to consider the combined effect of current and past on the coupling coordination degree, and avoid the problem of endogeneity as well as weak instrumental variables.

### Description of variables

Given the availability of data and previous results, the one-period lagged Xinjiang agriculture-tourism coupling coordination (L.coor) is chosen as the explanatory variable, while the explanatory variables are set as service capacity (se), human capital (ca), government support (gov), residents’ consumption level (con), industrial base (in) and market supply (ma).

Service capacity (se). Experience and participation are important factors in determining the success of integrated agri-tourism development. With the rapid development of technology, a convenient and smooth transportation and logistics environment are crucial to the satisfaction of tourists’ sense of experience. Service capacity, represented by transportation, warehousing, and postal services has a non-negligible impact on the degree of coordination of agri-tourism integration. We draw on previous research^35^ to measure the service capacity of a region regarding transport, storage, and postal services.

Human capital (ca). Higher human capital can drive labor productivity and directly contribute to sustained economic growth. To push the integration of rural industries, the key lies in talents, and the cultivation of talents is the foundation for the integration and revitalization of rural industries^36^. Thus, we add human capital, an important factor affecting coupling coordination, as an explanatory variable. We adopt the number of students enrolled in ordinary higher education institutions (HEIs) as the proxy variable for human capital, mainly because such students are an important component of the labor market, and their number reflects the level of education in the region.

Government support (gov). A good policy environment for the coordinated development of agri-tourism integration has a non-negligible safeguarding role. By giving policy preferences and incentives for agri-tourism integration projects and improving infrastructure at the grassroots level, the government’s financial support can be brought into play. Thus, we add policy support as an explanatory variable and integrate related studies^37^ to select general public budget expenditure as a proxy variable for policy support.

Residents’ consumption level (con). Residents’ consumption level is a key factor affecting the development of agri-tourism integration. In general, as residents’ discretionary income rises, so does the demand for service-oriented levels of travel and other services. Simultaneously, the main service objects of agricultural-tourism integration are the residents who live in the hustle and bustle of the city for a long time, Therefore, as a representative of residents’ consumption level, the changes in urban residents’ income will inevitably have a considerable impact on the integration of agriculture and tourism. We refer to Yang et al. who adopt urban residents’ disposable income as a representative indicator to characterize residents’ consumption level^38^.

Industrial base (in). Industrial base capability determines the overall quality, comprehensive strength, and core competitiveness of industries in a country and region^39^. We use the value-added of the tertiary sector to measure the industrial base, or whether the development of the tertiary sector can influence the degree of agri-tourism integration and coordination.

Market supply (ma). Market supply refers to the total quantity of a good or service available to consumers within a given market at a given time. In the tourism economy, the supply situation of the market affects the operation and development of the entire tourism market^40^. Therefore, we select the amount of fixed asset investment as a proxy variable for market supply. All variables are summarized in Table 4.

**Table 4:** Variables and their interpretation.

Variables	Variable explanation	Unit
Service capacity (se)	Transportation, storage, postal industry	Million yuan
Human Capital (ca)	No. of students enrolled in ordinary higher education institutions	People
Government Support (gov)	General fiscal public budget expenditure	Million yuan
Resident consumption level (con)	Per capita disposable income of urban residents	Yuan
Industrial Base (in)	Value-added of tertiary industry output	Billion
Market supply (ma)	Fixed assets investment amount	Million yuan

### Regression results

We use an econometric model to test the coupling coordination degree of agriculture and tourism and the interrelationships of service capacity, human capital, government support, residents’ consumption level, industrial base, and market supply in various cities and towns in Xinjiang from 2009 to 2018, and use the econometric software Stata14 to carry out the regression (see Table 5).

**Table 5:** Results of differential GMM estimation.

Variables	(1)	(2)	(3)	(4)	(5)	(6)
L.coord	0.886***	0.781***	0.785***	0.827***	0.798***	0.849***
	(168.19)	(39.26)	(51.94)	(23.49)	(20.82)	(17.86)
se	− 0.0164***	− 0.0150***	− 0.0141***	− 0.0128***	− 0.0129***	− 0.0151***
	(− 8.04)	(− 12.99)	(− 7.05)	(− 4.63)	(− 4.72)	(− 4.15)
ca		0.471***	0.493***	0.448***	0.501***	0.484***
		(8.41)	(10.08)	(6.24)	(5.46)	(5.40)
gov			− 0.0136**	− 0.0158**	− 0.0169**	− 0.0578
			(− 3.10)	(− 2.78)	(− 2.89)	(− 1.71)
con				0.0214**	0.0208*	0.0235*
				(2.68)	(2.30)	(2.08)
in					− 0.0151***	− 0.0199***
					(− 3.74)	(− 5.09)
ma						− 0.0408***
						(− 4.23)
Constant	0.0400***	0.0194	0.0150	0.00729	0.0111	0.0546
	(21.95)	(1.22)	(1.57)	(0.38)	(0.54)	(1.76)
Sample size	112	112	112	112	112	112
AR (1) test	0.149	0.0134	0.0133	0.0118	0.0153	0.0314
AR(2) test	0.2513	0.2923	0.2757	0.2586	0.3212	0.3005
Sargan test	0.8431	0.8594	0.8769	0.8783	0.8947	0.9909

To obtain robust test results, we gradually add six explanatory variables ((se), (ca), (gov), (con), (in), (ma)) to gradually test the influence of each factor on the degree of coupling coordination, and conduct the overall effect test.

From Table 5, the impact coefficient of service capacity on the degree of coupling coordination is significantly negative, indicating that the lagging development of Xinjiang’s transportation, storage, and postal industries constrains the improvement of coupling coordination. The possible reason for this is that although the transportation, storage and postal industry in Xinjiang is growing rapidly, the growth in output value lags behind the tertiary industry, which does not have a significant effect on the promotion of the tourism industry, and also weakens the impact on agritourism integration, displaying a negative correlation.

The coefficient of the effect of human capital on the degree of coupling coordination is significantly positive, which indicates that human capital has a strong positive contribution to the improvement of such degree. HE influences the economy through mediating variables such as science, technology, talent, and skills, and also contributes to agritourism integration in other ways such as consumption contribution and support for entrepreneurship. In other words, with the increase in the number of students enrolled in colleges and universities, the level of agricultural-tourism coupling coordination will continue to rise.

The effect of government support on coupling coordination is negative and insignificant. For Xinjiang, which is located in the western region, with limited financial resources, the government prefers to spend funds on areas with quick results and high returns. It is a relatively slow and continuous process for rural areas to rely on agricultural resources to develop tourism and to adjust and optimize the rural industrial structure; thus, despite the improvement of the fiscal expenditure inhibiting the development of agricultural-tourism integration, a negative impact remains.

Residents' consumption level is tested for significance at the 10% level, and this statistic indicates that urban residents' disposable income has a positive effect on the coupling and coordination of agriculture and tourism in Xinjiang.In fact, only when the disposable income of individuals increases, can it lead to consumption and the pursuit of higher-level demand. Alternatively, boosting disposable income to achieve high levels of demand will undoubtedly lead to the embedding of agriculture in tourism initiatives, which will in turn promote a high level of agritourism integration.

The influence coefficient of industrial base on the coupling coordination degree is significantly negative, indicating that the value-added of tertiary industry does not contribute significantly to the coupling coordination degree. Although the growth rate of the tertiary industry has been increasing, the development gap between regions and states is large, which restricts the development of the overall tertiary industry, thus inhibiting the influence of the industrial base on the coupled and coordinated development of agricultural and tourism integration. In other words, whether it is necessary to coordinate the ratio of primary, secondary, and tertiary industries in each state should be considered when formulating regional economic strategies.

Market supply is negatively correlated with coupling coordination degree. This is because the current fixed asset investment in Xinjiang is still in capital-intensive industries, infrastructure construction and other fields that have obvious driving effects on GDP. Moreover, the development of the service industry will not significantly enhance its efficiency, but will have a squeezing effect, which also leads to a negative performance of the effect of market supply on the degree of coupling coordination.

## Conclusion and policy implications

### Conclusions

We apply the coupling coordination model to measure and analyze the development level of agriculture-tourism integration in various cities and towns in Xinjiang from 2009 to 2018, and empirically investigate the main factors affecting the degree of coupling coordination between agriculture and tourism using GMM estimation. The level of agricultural and tourism development in all cities and towns in Xinjiang exhibited an upward trend, but the coupling degree and the coupling coordination degree of agricultural-tourism integration in all cities and towns displayed significant spatial differentiation characteristics. Meanwhile, among the influencing factors of the coordinated development of agricultural and tourism integration, human capital, level of residents’ consumption, and degree of coupling coordination are positively correlated, while service capacity, government support, industrial foundation, and market supply are negatively correlated with the degree of coupling coordination.

### Policy implications

**Figure 5:**
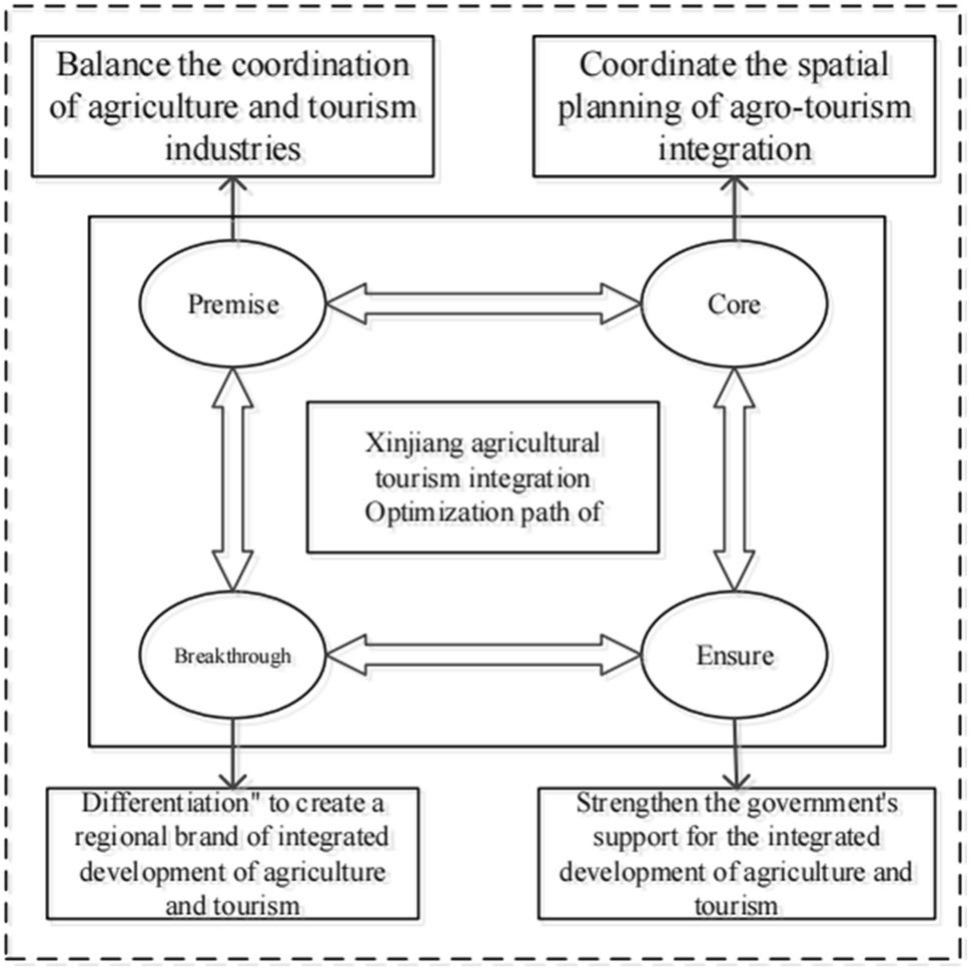
Schematic diagram of the enhancement path for the integrated development of agriculture and tourism in Xinjiang.

The spatial differentiation pattern in the development process of Xinjiang’s agritourism integration hinders the coordinated development of its overall agritourism integration. Therefore, we propose the following enhancement paths (see Fig. 5).

1. Balance the coordination of agriculture and tourism industries. Attaching importance to the coordinated relationship between agriculture and tourism, not only to achieve “tourism + ,” but also to ensure “agriculture + ,” combining agricultural and rural development with the establishment, promotion, and operation of the tourism industry, and realizing a high level of development in the integration of agriculture and tourism.
2. Coordinate the spatial planning of agri-tourism integration. Cooperation between regions should be strengthened to enhance regional linkages, especially in the northern border region, which, on the basis of ensuring its own development, has taken the initiative to share its experience with the southern border region and to channel specialized talents, and drive the coordinated development of agri-tourism integration across the region.
3. Differentiation to create a regional brand of integrated development of agriculture and tourism. The Northern Xinjiang region should focus on leveraging the advantages of tourism resource endowment, filling the gaps in agricultural foundation, and promoting agriculture through tourism. For the southern border regions, agricultural scale and specialization can be formed, special planting bases can be established, and farmers can be encouraged to participate in the operation, so as to promote the income of farmers and herdsmen, thereby driving the development of the tourism.
4. Strengthen the government’s support for the integrated development of agriculture and tourism. Governments should take effective measures in accordance with local conditions, increase the construction of rural infrastructure projects in the southern border areas, improve transportation, warehousing, and other infrastructures, and enhance the service capacity in the integrated development of agri-tourism in these areas. Additionally, the government should also guide the Southern Xinjiang region to develop its own characteristic advantages, such as integrating products with unique local characteristics such as ethnic culture, folk customs, and special agricultural products, so as to make regional agri-tourism products more diversified and characteristic.

## Data Availability

The datasets used and analyzed during the current study are available from the corresponding author upon reasonable request.
